# Supplementary material for: Predictive Modeling for Suicide-Related Outcomes and Risk Factors among Patients with Pain Conditions: A Systematic Review
Source: J Clin Med. 2022 Aug 17;11(16):4813. doi: 10.3390/jcm11164813 (PMC9409905; doi:10.3390/jcm11164813)
Supplement: Supplementary file 1 [file jcm-11-04813-s001.zip › jcm-1873440-supplementary.pdf]

Supplementary Online Content

Table S1. Suicide Prediction Modeling Systematic Review Search Queries (Searches Were Conducted on December 9th, 2020)

Table S2. Characteristics of Included Studies (n=87)

Table S3. Summary of Individual Risk Factors Identified from Less Than 3 Studies by Data Source for Identification

Table S4. Identified Risk Factors in Each Risk Factor Category

Table S1. Suicide Prediction Modeling Systematic Review Search Queries (Searches Were Conducted on December 9th, 2020)

Sources	Search strategies
**PubMed**	("Chronic Pain"[MeSH Terms] OR "Chronic Pain"[Title/Abstract] OR ("chronic"[Title/Abstract] AND "pain*"[Title/Abstract]) OR "Pain"[Title/Abstract] OR "Pain"[MeSH Terms]) AND ("Suicide"[Mesh] OR suicid*[tiab] OR "Suicidal Ideation"[Mesh] OR "Suicide, Attempted"[Mesh] OR Parasuicide*[tiab]) AND ("Adult"[Mesh] OR adult*[tiab] OR elder*[tiab] OR aged[tiab]) AND ((risk*[tiab] OR screen*[tiab] OR assessment*[tiab] OR predict*[tiab] OR "Risk Assessment"[Mesh] OR "Risk Assessment"[Mesh] OR "Mass Screening"[Mesh]) OR ("instrumentation" [Subheading] OR instrumentation*[tiab] OR instrument*[tiab] OR tool*[tiab] OR algorithm*[tiab] OR model*[tiab] OR "machine learning"[tiab] OR "decision tree"[tiab] OR "decision trees"[tiab] OR "decision theory"[tiab] OR "decision theories"[tiab] OR "data mining"[tiab] OR "text mining"[tiab] OR "Machine Learning"[Mesh] OR "Models, Statistical"[Mesh] OR "Decision Theory"[Mesh] OR "Decision Trees"[Mesh] OR "Algorithms"[Mesh] OR "Data Mining"[Mesh] OR "Predictive Value of Tests"[Mesh] OR "Predictive Value of Tests"[tiab] OR "predictive value"[tiab])); Filter: English, from 2000 - 2020
**CINAHL**	(TI ((MH "Suicide+") OR suicid* OR "Suicidal Ideation" OR Parasuicide*) OR AB (suicid* OR "Suicidal Ideation" OR Parasuicide*)) AND ((MM "Chronic Pain") OR (MH "Pain") OR TI (("Chronic Pain" OR (chronic AND pain*) OR Pain OR)) OR AB (("Chronic Pain" OR (chronic AND pain*) OR Pain OR)) AND (TI (adult* OR aged OR elder*) OR AB (adult* OR aged OR elder*) OR (MH "Adult+")) AND (((MH "Risk Assessment") OR (MH "Prediction Models")) OR TI (risk* OR screen* OR assessment* OR predict* OR "Risk Assessment" OR "Risk Assessment" OR "Mass Screening) OR AB (risk* OR screen* OR assessment* OR predict* OR "Risk Assessment" OR "Risk Assessment" OR "Mass Screening) OR ((MH "Machine Learning") OR (MH "Models, Statistical+") OR (MH "Predictive Value of Tests") OR (MH "Decision Trees+")) OR TI (instrumentation* OR instrument* OR tool* OR algorithm* OR model* OR "machine learning" OR "decision tree" OR "decision trees" OR "decision theory" OR "decision theories" OR "data mining" OR "text mining" OR "Predictive Value of Tests" OR "predictive value" OR “statistical model”) OR AB (instrumentation* OR instrument* OR tool* OR algorithm* OR model* OR "machine learning" OR "decision tree" OR "decision trees" OR "decision theory" OR "decision theories" OR "data mining" OR "text mining" OR "Predictive Value of Tests" OR "predictive value" OR “statistical model”)); Limiters - Published Date: 20000101-20201231 Narrow by Language: - english
**Cochrane Library**	((suicid* OR "Suicidal Ideation" OR Parasuicide*):ti,ab,kw) AND ((("Chronic Pain" OR (chronic AND pain*) OR Pain OR veteran*)):ti,ab,kw) AND ((adult* OR aged OR elder*):ti,ab,kw) AND (((risk* OR screen* OR assessment* OR predict* OR "Risk Assessment" OR "Risk Assessment" OR "Mass Screening"):ti,ab,kw) OR ((instrumentation* OR instrument* OR tool* OR algorithm* OR model* OR "machine learning" OR "decision tree" OR "decision trees" OR "decision theory" OR "decision theories" OR "data mining" OR "text mining" OR "Predictive Value of Tests" OR "predictive value" OR “statistical model”):ti,ab,kw)); Restricted to Cochrane Reviews

Table S1. (Continued)

Sources	Search strategies
**Embase**	('Article'/it OR 'Article in Press'/it OR 'Conference Abstract'/it OR 'Conference Paper'/it OR 'Conference Review'/it) AND (('chronic pain'/exp OR 'chronic intractable pain':ti,ab OR 'chronic pain':ti,ab OR 'pain, chronic':ti,ab OR 'pain'/de OR 'acute pain':ti,ab OR 'deep pain':ti,ab OR 'lightning pain':ti,ab OR 'nocturnal pain':ti,ab OR 'pain':ti,ab OR 'pain response':ti,ab OR 'pain syndrome':ti,ab OR 'treatment related pain':ti,ab OR (chronic:ti,ab AND pain:ti,ab)) AND ('suicide'/exp OR 'completed suicide':ti,ab OR 'self killing':ti,ab OR 'suicidal poisoning':ti,ab OR 'suicide':ti,ab OR 'suicide, completed':ti,ab OR suicid*:ti,ab OR 'suicide attempt'/exp OR 'attempted suicide':ti,ab OR 'parasuicide':ti,ab OR 'suicidal attempt':ti,ab OR 'suicide attempt':ti,ab OR 'suicide, attempted':ti,ab OR 'tentamen suicidi':ti,ab) AND ('adult'/exp OR 'adult':ti,ab OR 'adults':ti,ab OR 'grown-ups':ti,ab OR 'grownup':ti,ab OR 'grownups':ti,ab OR adult*:ti,ab OR elder*:ti,ab) AND (('mass screening'/exp OR 'health screening':ti,ab OR 'health screening program':ti,ab OR 'health screening programme':ti,ab OR 'longitudinal health screening program':ti,ab OR 'longitudinal health screening programme':ti,ab OR 'mass screening':ti,ab OR 'population screening':ti,ab OR 'screening, mass':ti,ab OR 'risk assessment'/exp OR 'assessment, safety':ti,ab OR 'risk adjustment':ti,ab OR 'risk analysis':ti,ab OR 'risk assessment':ti,ab OR 'risk evaluation':ti,ab OR 'safety assessment':ti,ab OR risk*:ti,ab OR screen*:ti,ab OR assessment*:ti,ab) OR (instrumentation*:ti,ab OR 'machine learning'/exp OR 'learning machine':ti,ab OR 'learning machines':ti,ab OR 'machine learning':ti,ab OR 'decision tree'/exp OR 'decision tree':ti,ab OR 'decision trees':ti,ab OR 'statistical model'/exp OR 'likelihood functions':ti,ab OR 'linear model':ti,ab OR 'linear models':ti,ab OR 'logistic models':ti,ab OR 'models, statistical':ti,ab OR 'statistic model':ti,ab OR 'statistical model':ti,ab OR 'statistical models':ti,ab OR 'statistics model':ti,ab OR 'predictive value'/exp OR 'negative predictive value':ti,ab OR 'positive predictive value':ti,ab OR 'predictive value':ti,ab OR 'predictive value of tests':ti,ab OR 'data mining'/exp OR 'data mining':ti,ab OR 'datamining':ti,ab OR 'text mining'/exp OR 'text mining':ti,ab OR instrumentation*:ab,ti OR instrument*:ab,ti OR tool*:ab,ti OR algorithm*:ab,ti OR model*:ab,ti OR 'machine learning':ab,ti OR 'decision tree':ab,ti OR 'decision trees':ab,ti OR 'decision theory':ab,ti OR 'decision theories':ab,ti OR 'data mining':ab,ti OR 'text mining':ab,ti OR 'predictive value of tests':ab,ti OR 'predictive value':ab,ti OR 'statistical model':ab,ti)) AND ([english]/lim AND [2000-2021]/py))
**ProQuest Thesis and Dissertations**	(ab(suicid* OR "Suicidal Ideation" OR Parasuicide*) OR ti(suicid* OR "Suicidal Ideation" OR Parasuicide*)) AND (ti("Chronic Pain" OR (chronic AND pain*) OR Pain*) OR ab("Chronic Pain" OR (chronic AND pain*) OR Pain*)) AND (ti(adult* OR aged* OR elder*) OR ab(adult* OR aged* OR elder*)) AND ((ab(risk* OR screen* OR assessment* OR predict* OR "Risk Assessment" OR "Risk Assessment" OR "Mass Screening") OR ti(risk* OR screen* OR assessment* OR predict* OR "Risk Assessment" OR "Risk Assessment" OR "Mass Screening")) OR (ab(instrumentation* OR instrument* OR tool* OR algorithm* OR model* OR "machine learning" OR "decision tree" OR "decision trees" OR "decision theory" OR "decision theories" OR "data mining" OR "text mining" OR "Predictive Value of Tests" OR "predictive value" OR “statistical model”) OR ti(instrumentation* OR instrument* OR tool* OR algorithm* OR model* OR "machine learning" OR "decision tree" OR "decision trees" OR "decision theory" OR "decision theories" OR "data mining" OR "text mining" OR "Predictive Value of Tests" OR "predictive value" OR “statistical model”))) AND yr(2000-2021)

Table S1. (Continued)

**Psych Info**	(TI (suicid* OR "Suicidal Ideation" OR Parasuicide*) OR AB (suicid* OR "Suicidal Ideation" OR Parasuicide*)) AND (TI (("Chronic Pain" OR (chronic AND pain*) OR Pain)) OR AB (("Chronic Pain" OR (chronic AND pain*) OR Pain))) AND (TI (adult* OR aged OR elder*) OR AB (adult* OR aged OR elder*)) AND ((TI (risk* OR screen* OR assessment* OR predict* OR "Risk Assessment" OR "Risk Assessment" OR "Mass Screening") OR AB (risk* OR screen* OR assessment* OR predict* OR "Risk Assessment" OR "Risk Assessment" OR "Mass Screening")) OR (TI (instrumentation* OR instrument* OR tool* OR algorithm* OR model* OR "machine learning" OR "decision tree" OR "decision trees" OR "decision theory" OR "decision theories" OR "data mining" OR "text mining" OR "Predictive Value of Tests" OR "predictive value" OR “statistical model”) OR AB (instrumentation* OR instrument* OR tool* OR algorithm* OR model* OR "machine learning" OR "decision tree" OR "decision trees" OR "decision theory" OR "decision theories" OR "data mining" OR "text mining" OR "Predictive Value of Tests" OR "predictive value" OR “statistical model”))); Limiters - Publication Year: 2000-2020, Expanders - Apply equivalent subjects, Narrow by Language: - english, Search modes - Find all my search terms
**Web of Science**	(TOPIC: (("Chronic Pain" OR "Chronic Pain" OR ("chronic" AND "pain*") OR "Pain" OR "Pain")) Indexes=SCI-EXPANDED, SSCI, A&HCI, ESCI Timespan=All years) AND (TOPIC: ("Suicide" OR suicid* OR "Suicidal Ideation" OR "Suicide, Attempted" OR Parasuicide*) Indexes=SCI-EXPANDED, SSCI, A&HCI, ESCI Timespan=All years) AND (TOPIC: ("Adult" OR adult* OR elder* OR aged) Indexes=SCI-EXPANDED, SSCI, A&HCI, ESCI Timespan=All years) AND ((TOPIC: (risk* OR screen* OR assessment* OR predict* OR "Risk Assessment" OR "Risk Assessment" OR "Mass Screening") Indexes=SCI-EXPANDED, SSCI, A&HCI, ESCI Timespan=All years) OR (TOPIC: ("instrumentation" OR instrumentation* OR instrument* OR tool* OR algorithm* OR model* OR "machine learning" OR "decision tree" OR "decision trees" OR "decision theory" OR "decision theories" OR "data mining" OR "text mining" OR "Machine Learning" OR "Models, Statistical" OR "Decision Theory" OR "Decision Trees" OR "Algorithms" OR "Data Mining" OR "Predictive Value of Tests" OR "Predictive Value of Tests" OR "predictive value") Indexes=SCI-EXPANDED, SSCI, A&HCI, ESCI Timespan=All years)); Refined by: PUBLICATION YEARS: (2020 OR 2012 OR 2005 OR 2019 OR 2011 OR 2004 OR 2018 OR 2010 OR 2003 OR 2017 OR 2009 OR 2002 OR 2016 OR 2008 OR 2001 OR 2015 OR 2007 OR 2000 OR 2014 OR 2006 OR 2013) AND DOCUMENT TYPES: (ARTICLE OR EARLY ACCESS OR MEETING ABSTRACT OR PROCEEDINGS PAPER) AND LANGUAGES: (ENGLISH) Indexes=SCI-EXPANDED, SSCI, A&HCI, ESCI Timespan=All years
**SCOPUS**	(TITLE-ABS (suicid* OR "Suicidal Ideation" OR parasuicide*)) AND (TITLE-ABS ("Chronic Pain" OR (chronic AND pain*) OR pain)) AND ((TITLE-ABS (risk* OR screen* OR assessment* OR predict* OR "Risk Assessment" OR "Risk Assessment" OR "Mass Screening")) OR (TITLE-ABS (instrumentation* OR instrument* OR tool* OR algorithm* OR model* OR "machine learning" OR "decision tree" OR "decision trees" OR "decision theory" OR "decision theories" OR "data mining" OR "text mining" OR "Predictive Value of Tests" OR "predictive value" OR "statistical model"))) AND (TITLE-ABS-KEY (adult* OR aged OR elder*)) AND (LIMIT-TO (DOCTYPE , "ar") OR LIMIT-TO (DOCTYPE , "cp")) AND (LIMIT-TO (PUBYEAR , 2021) OR LIMIT-TO (PUBYEAR , 2020) OR LIMIT-TO (PUBYEAR , 2019) OR LIMIT-TO (PUBYEAR , 2018) OR LIMIT-TO (PUBYEAR , 2017) OR LIMIT-TO (PUBYEAR , 2016) OR LIMIT-TO (PUBYEAR , 2015) OR LIMIT-TO (PUBYEAR , 2014) OR LIMIT-TO (PUBYEAR , 2013) OR LIMIT-TO (PUBYEAR , 2012) OR LIMIT-TO (PUBYEAR , 2011) OR LIMIT-TO (PUBYEAR , 2010) OR LIMIT-TO (PUBYEAR , 2009) OR LIMIT-TO (PUBYEAR , 2008) OR LIMIT-TO (PUBYEAR , 2007) OR LIMIT-TO (PUBYEAR , 2006) OR LIMIT-TO (PUBYEAR , 2005) OR LIMIT-TO (PUBYEAR , 2004) OR LIMIT-TO (PUBYEAR , 2003) OR LIMIT-TO (PUBYEAR , 2002) OR LIMIT-TO (PUBYEAR , 2001) OR LIMIT-TO (PUBYEAR , 2000)) AND (LIMIT-TO (LANGUAGE , "English"))

Table S2. Characteristics of Included Studies (n=87)

Author, year	Country	Study Design	Type of Data Sources	Study Population[a]	Total # Pts	Cancer Pts	Suicide-related Outcome(s)	Statistical methods	Age (Mean/ Median*)	Female %	Outcome Proportion	Identified Risk Factor Categories[b]
Abdelghani, 2020[1]	USA	Cross-sectional	Nationwide questionnaire	Pts with a history of chronic pain	5,301	No	SI & SA	Logistic regression	42 (SI), 40 (SA)	25 (SI), 42 (SA)	18.6% (SI), 7.0% (SA)	3, 10, 22, 26
Akechi, 2002[2]	Japan	Cross-sectional	Single site questionnaire	Pts with newly diagnosed unresectable non-small cell lung carcinoma	89	Yes	SI	Logistic regression	61	28	15.0%	5, 8
Akechi, 2020[3]	Japan	Cross-sectional	Single site questionnaire	Pts with multiple myeloma	79	Yes	SI	Logistic regression	66	49	12.6%	6, 8, 25
Alacreu-Crespo, 2020[4]	France	Prospective cohort	Single site questionnaire	Pts with depression	843	No	SB	Logistic regression	42	68	23.1%	27
Andrijic, 2014[5]	Bosnia and Herzegovina	Cross-sectional	Single site questionnaire	Epilepsy inpatients and outpatients	50	No	SI	Logistic regression	39	50	38.0%	16, 25
Applebaum, 2019[6]	USA	Retrospective cohort	State and national administrative data, NDI	New Mexico workers receiving workers' compensation benefits	100,806	No	SD	Competing risks regression	Age 25-34: 29%	38	5.8%	16
Ashrafioun, 2016[7]	USA	Retrospective cohort	Integrated VHA, Surveillance data	US veterans with an alcohol use disorder who had an inpatient hospitalization	13,047	No	SA	Logistic regression	Age 35-54: 47%	5	7.2% (Prior year SA), 1.7% (Following year SA)	1, 5, 8
Ashrafioun, 2019[8]	USA	Retrospective cohort	Integrated VHA, Surveillance data	US veterans in VHA pain specialty services	221,817	No	SA	Cox proportional hazards model	Age 50-64: 43%	10	1,023/100,000 PY	3, 5,8, 10, 11, 13, 21, 24, 26
Berhane, 2018[9]	Ethiopia	Cross-sectional	Single site questionnaire	Outpatients with and without migraines	1,060	No	SB	Logistic regression	36	60	15.1%	12
Blakey, 2018[10]	USA	Cross-sectional	Multisite questionnaire	Post-9/11 US veterans with chronic pain	667	No	SI	Logistic regression	38	19	19.04% (SI), 12.29% (Violent impulses)	8, 21, 22, 26
Braden, 2008[11]	USA	Retrospective cohort	Nationwide questionnaire	Pts with self-reported pain conditions	5,692	No	SB	Logistic regression	Age 18-44: 43-64%	47-59	19.3% (SI); 7.2% (suicide plan); 6.6% (SA)	5
Breslau, 2012[12]	USA	Prospective cohort	Community questionnaire	Migraine and headache pts and community sample of controls	1,186	No	SA	Logistic regression	41	79	5.6%	3, 8, 12

Table S2. (Continued)

Author, year	Country	Study Design	Type of Data Sources	Study Population[a]	Total # Pts	Cancer Pts	Suicide-related Outcome(s)	Statistical methods	Age (Mean/ Median*)	Female %	Outcome Proportion	Identified Risk Factor Categories[b]
Brown, 2020[13]	USA	Cross-sectional	Multisite questionnaire	Pts with chronic nonmalignant pain on LTOT who did not develop an opioid use disorder	496	No	SB	GEE models	55	34	N/A	19
Bryant, 2016[14]	Australia	Prospective cohort	Multisite questionnaire	Traumatically injured pts	1,129	No	SD	Logistic regression	Age 35-44: 23.7-27.7%	27-31	7.9%	5, 16, 27
Caceda, 2014[15]	USA	Cross-sectional	Multisite questionnaire	Depressed pts and healthy controls	82	No	SI & SB	Logistic regression	36-46	50-65	26.83% (SI); 24.39% (SA)	8, 27
Calandre, 2015[16]	Spain	Cross-sectional	Single site questionnaire	Pts with FM	373	No	SI	Linear regression	49	95	48.0%	3, 5, 8, 9, 24
Campbell,2015[17]	Australia	Cross-sectional	Community questionnaire	Community sample of chronic non-cancer pain pts prescribed opioids for pain	978	No	SI	Logistic regression	58	65	19.7% (SI); 19.6% (SA)	15
Campbell,2016[18]	Australia	Cross-sectional	Community questionnaire	Community based people prescribed opioids for chronic noncancer pain	1,514	No	SI	Logistic regression	58	66	19.7% (SI); 19.6% (SA)	5, 8, 11
Campbell,2020[19]	Australia	Case-control	Nationwide questionnaire	Intentional deaths among chronic chronic non-cancer pain pts	2,590	No	SD	Logistic regression	46	24	N/A	1, 2, 4, 5, 8, 13, 14, 17, 18, 23, 25, 27
Cheatle, 2014[20]	USA	Cross-sectional	Single site EMR	Pts with chronic non-cancer pain referred to a behaviorally based pain program	466	No	SI	Logistic regression	48	60	28.1%	16
Copeland,2014[21]	USA	Retrospective cohort	VHA	VHA pts who underwent major surgery	89,995	No	SB & SI	Cox proportional hazards model	64	4	3.2%	1, 2, 5, 8, 10, 11, 15, 16, 17, 21, 22, 25
Demidenko,2017[22]	USA	Case-control	VHA	VHA pts whose clinicians discontinued them from LTOT	509	No	SI & SSV	Logistic regression	55	5	9.2% (SI); 2.4% (SA)	21, 22, 27
Edwards, 2006[23]	USA	Cross-sectional	Single site questionnaire	Pts seeking treatment for chronic pain	1,512	No	SI	Linear regression	46	60	32.0%	1, 3, 5, 8, 16, 19

Table S2. (Continued)

Author, year	Country	Study Design	Type of Data Sources	Study Population[a]	Total # Pts	Cancer Pts	Suicide-related Outcome(s)	Statistical methods	Age (Mean/ Median*)	Female %	Outcome Proportion	Identified Risk Factor Categories[b]
Edwards, 2007[24]	USA	Prospective cohort	Multisite questionnaire	Survivors of major burns	128	No	SI	Logistic regression	41	30	17.2% (Passive SI); 14.8% (Active SI)	5
Fishbain,2009[c] [25]	USA	Cross-sectional	Single site questionnaire	Chronic LBP pts who smokes	81	No	SI	Logistic regression	41	37	28.4%	8, 16
Fishbain,2011[c] [26]	USA	Cross-sectional	Community questionnaire, Multisite questionnaire	Rehabilitation pain pts	2,264	No	SI	Logistic regression	38 (CH), 44 (CP), 41 (RP), 36 (RAP), 40 (RCP)	57 (CH), 55 (CP), 59 (RP), 55 (RAP), 56 (RCP)	1.9% (CH), 3.2% (CP), 3.6% (RP), 4.0% (RAP), 4.4% (RCP)	11, 16
Fishbain,2012[c] [27]	USA	Cross-sectional	Community questionnaire, Multisite questionnaire	Rehabilitation pain pts	2,264	No	SI[d]	Logistic regression	N/A	N/A	19.4% (CH), 15.7% (CP), 22.7% (RAP), 18.5% (RCP)	11, 16
Fishbain,2012[c] [28]	USA	Cross-sectional	Community questionnaire, Multisite questionnaire	Rehabilitation pain pts	2,264	No	SB	Logistic regression	N/A	N/A	N/A	5, 8, 11, 15, 16, 19, 21, 23
Fishbain, 2016[29]	USA	Cross-sectional	Community questionnaire, Multi-site questionnaire	Pts in rehabilitation facilities and community subjects	2,264	No	SB	Logistic regression	36 (Acute pain); 40 (Chronic pain)	55 (Acute pain); 56 (Chronic pain)	18.52-33.33% (Acute pain); 17.65-32.35% (Chronic pain)	20
Fonda, 2015[30]	USA	Retrospective cohort	VHA	Veterans, with and without Traumatic brain injury	261,304	No	SA	Cox proportional hazards model	29	16	74.25 per 100,000	7
Frumkin, 2020[31]	USA	Cross-sectional	Community questionnaire	Bereaved adults	135	No	SI & SA	Logistic regression	47	62	20% (Passive SI); 18.5% (Lifetime SA)	27
Fuller-Thomson, 2020[32]	Canada	Cross-sectional	Nationwide questionnaire	Pts with migraine headaches	2,223	No	SA	Logistic regression	N/A	N/A	8.7% (Migraine); 2.3% (No migraine)	3, 8, 16, 19, 25, 26
Gensichen, 2010[33]	Germany	Cross-sectional	Single site EMR, Single site questionnaire	Pts with major depression	626	No	SI	Logistic regression	50	76	56.4%	5, 8
Harnod, 2018[34]	Taiwan	Retrospective cohort	Nationwide claims	Pts with RM and SM	35,090	No	SA	Cox proportional hazards model	45	74	2.6 per 100,000 PY (SM); 1.91 per 100,000 PY (RM)	12

Table S2. (Continued)

Author, year	Country	Study Design	Type of Data Sources	Study Population[a]	Total # Pts	Cancer Pts	Suicide-related Outcome(s)	Statistical methods	Age (Mean/ Median*)	Female %	Outcome Proportion	Identified Risk Factor Categories[b]
Hashash, 2019[35]	USA	Cross-sectional	Single site chart review, Single site EMR, Single site questionnaire	inflammatory bowel diseases outpatients	1,352	No	SI	Logistic regression	43	45	5.3%	2, 8
Ilgen, 2010[36]	USA	Cross-sectional	VHA, Multi-site questionnaire, NDI	Veterans with pain and/or mental illness	260,254	No	SD	Cox proportional hazards model	Age ≥65: 47.76%	5	58.24 per 100,000 PY	19
Ilgen, 2013[37]	USA	Retrospective cohort	VHA	All individuals receiving VHA services	4,863,086	No	SD	Cox proportional hazards model	Age 50-59: 23.9%	8	0.01%	12, 18, 27
Ilgen, 2016[38]	USA	Case-control	VHA	VHA pts with chronic pain conditions treated with opioids, who later died by suicide	123,946	No	SD	Cox proportional hazards model	Age 50-59: 31-33%	7	44.4 per 100,000 PY	13
Im, 2015[39]	USA	Cross-sectional	VHA	Veteran pts prescribed opioids on a chronic basis	487,462	No	SA	Logistic regression	Age >55: 46-70%	7-10	7.2% (CSA opioid); 8.3% (LA opioid)	13, 15
Jakubczyk, 2016[40]	USA	Cross-sectional	Single site questionnaire	Pts with alcohol dependence	366	No	SB	Logistic regression	41 (History of SA), 45 (No history of SA)	26-27	32.2%	8, 19
Jang, 2020[41]	South Korea	Case-control	Nationwide claims	Spine fracture pts older than 65 years	31,357 (cases), 62,714 (controls)	No	SD	Generalized linear model	72	79	116 per 100,000 PY	18
Jeon, 2016[42]	South Korea	Cross-sectional	Multisite questionnaire	MDD outpatients	811	No	SB	Linear regression	46	72	N/A	18
Jha, 2020[43]	USA	Cross-sectional	Multisite questionnaire	MDD pts with and without anger attacks	309 (MDD), 40 (healthy controls)	No	SI	Generalized linear model	37	64-66	N/A	15
Jimenez-Rodriguez, 2014[44]	Spain	Case-control	Single site questionnaire	Pts with FM or LBP lasting ≥3 months	126	No	SI	Logistic regression	55 (FM), 50 (LBP), 51 (Control)	93 (FM), 56 (LBP), 72 (Control)	54.6% (FM); 18.8% (LBP); 4% (Control)	9, 18

Table S2. (Continued)

Author, year	Country	Study Design	Type of Data Sources	Study Population[a]	Total # Pts	Cancer Pts	Suicide-related Outcome(s)	Statistical methods	Age (Mean/ Median*)	Female %	Outcome Proportion	Identified Risk Factor Categories[b]
Johnson, 2020[45]	USA	Cross-sectional	Single site chart review	Veterans with cancer who were referred for psychology services	175	Yes	SI	Logistic regression	62	22	25.1%	8
Kanzler, 2012[46]	USA	Retrospective cohort	Single site chart review, Single site questionnaire	Pts referred for pain related health complaints	113	No	SI	Logistic regression	42	65	15.0%	20
Lan, 2016[47]	Taiwan	Cross-sectional	National claims	Pts with incident FM	95,150	No	SB	Cox proportional hazards model	46	58	4.16/10,000 PY	9
Levi-Belz, 2014[48]	Israel	Case-control	Single site questionnaire, Single site EMR, Structured interview	Pts admitted to a psychiatric hospital	336	No	SA	MANOVA and hierarchical regression	39 (MSSA), 39 (MNSSA), 41 (PC), 39 (HC)	44 (MSSA), 56 (MNSSA), 70 (PC), 45 (HC)	57.7% (MSSA & MNSSA)	15, 16
Levi-Belz, 2018[49]	Israel	Case-control	Single site questionnaire	Pts admitted to a psychiatric hospital	241	No	SA	MANOVA and hierarchical regression	39(MSSA), 39 (MNSSA), 41 (HC)	49 (MSSA), 54 (MNSSA), 48 (HC)	80.5% (MSSA & MNSSA)	17
Levi-Belz, 2019[50]	Israel	Case-control	Single site questionnaire, Single site EMR, Structured interview	Pts admitted to a psychiatric hospital	338	No	SA	Logistic regression	39 (MSSA), 39 (MNSSA), 41 (PC), 39 (HC)	44 (MSSA), 56 (MNSSA), 30 (PC), 43 (HC)	57.4% (MSSA & MNSSA)	15
Li, 2014[51]	China	Cross-sectional	Single site questionnaire, Structured interview	Outpatients with major depressive episodes	111	No	SI	Stepwise regression analyses	29 (Case), 34 (Control)	71 (Case), 60 (Control)	N/A	19
Liu, 2015[52]	Taiwan	Cross-sectional	Single site questionnaire	Pts with migraine seen at headache clinic	1,318	No	SI & SA	Logistic regression	43	82	27.3% (SI), 6.9% (SA)	3, 8, 9, 12, 24
Lopez-Morinigo, 2018[c53]	UK	Retrospective cohort	Single site EMR	Pts seen in a comprehensive pain clinic	13,758	No	SD	Cox proportional hazards model	41 (SD); 41 (Non-suicide deaths)	31 (SD); 43 (Non-suicide deaths)	100/100,000 PY	16
Margari, 2014[54]	Italy	Cross-sectional	Single site questionnaire	Pts who were referred to the University Hospital of Bari	208	No	SD	Logistic regression	61 (Case), 62 (Control)	31 (Case), 45 (Control)	N/A	5, 8, 15

Table S2. (Continued)

Author, year	Country	Study Design	Type of Data Sources	Study Population[a]	Total # Pts	Cancer Pts	Suicide-related Outcome(s)	Statistical methods	Age (Mean/ Median*)	Female %	Outcome Proportion	Identified Risk Factor Categories[b]
Mazereeuw, 2020[55]	Canada	Case-control	National claims	Opioid-related suicides involving either oxycodone or hydromorphone	438	No	SD	Logistic regression	49* (Case), 45* (Control)	49 (Case), 38 (Control)	26.5%	13
McCracken, 2018[56]	UK	Cross-sectional	Single site questionnaire	Chronic pain pts	424	No	SI	Logistic regression	46	71	45.7%	8, 16
McKernan, 2018[c57]	USA	Case-control	Single site EMR	Pts with FM	8,879	No	SI & SA	Bootstrapped L-1 penalized regression	45 (SA cases), 57 (SA controls); 50 (SI cases), 57 (SI controls)	91 (SA cases), 90 (SA controls); 84 (SI cases), 91 (SI controls)	0.4% (SA); 1.1% (SI)	15, 16, 26
Mitchell, 2017[58]	Australia	Retrospective cohort	Multisite EMR	Pts with physical illness	12,111 (Case), 474,158 (Control)	Both	SB	Logistic regression	≥50	56 (Case), 59 (Control)	N/A	5, 6, 17, 23
Noyman-Veksler, 2017[59]	Israel	Cross-sectional	Multisite questionnaire	Pts with chronic pain seen in outpatient pain clinics	165	No	SI	Logistic regression	49	53	14.5%	8, 19, 20
Orr, 2020[60]	USA	Cross-sectional	Multisite questionnaire	Pts who used opioids and had self-reported moderate to severe pain	431	No	SI	Conditional process modeling	39	75	N/A	26
Ozdemiroglu, 2017[61]	Turkey	Cross-sectional	Single site chart review, Multisite questionnaire	Pts seen in outpatient oncology clinics with cancer	117	Yes	SI	Logistic regression	57	49	N/A	15, 18
Panagioti, 2012[62]	UK	Cross-sectional	Single site questionnaire	Pts who experienced a serious traumatic event	95	No	SB	MANOVA	30 (PTSD), 27 (Trauma)	76 (PTSD), 76 (Trauma)	74% (PTSD), 26.8% (Trauma)	21
Park, 2012[63]	South Korea	Cross-sectional	Community questionnaire	Pts with medically unexplained pain	6,510	No	SI	Logistic regression	18-64	76 (Case), 49 (Control)	9.4% (Case), 2.8% (Control)	5
Park, 2016[64]	South Korea	Cross-sectional	Single site questionnaire, Structured interview	Advanced outpatient cancer pts (colon, breast, cervical, lung)	457	Yes	SB	Linear regression	Age 50-59: 40%	75	40.6%	3, 16, 18

Table S2. (Continued)

Author, year	Country	Study Design	Type of Data Sources	Study Population[a]	Total # Pts	Cancer Pts	Suicide-related Outcome(s)	Statistical methods	Age (Mean/ Median*)	Female %	Outcome Proportion	Identified Risk Factor Categories[b]
Park, 2019[65]	South Korea	Cross-sectional	Nationwide questionnaire	Pts with a history of pain (multiple or single somatic pain)	12,532	No	SA	Logistic regression	45 (MSP), 43 (SSP), 45 (No pain)	77 (MSP), 72 (SSP), 58 (No pain)	56.8% (MSP), 35.8% (SSP), 19.3% (No pain)	5
Park, 2020[66]	South Korea	Cross-sectional	Nationwide questionnaire	Pts reported as having one or more symptoms of temporomandibular disorder	4,650	No	SI	Logistic regression	19-59	100	N/A	18
Racine, 2014[67]	Canada	Cross-sectional	Multisite questionnaire, Structured interview	Chronic pain pts recruited before their first appointment	88	No	SI & SA	Logistic regression	53	66	24.0%	2, 4, 15, 16, 24, 25
Racine, 2017[68]	Canada	Cross-sectional	Multisite questionnaire	Pts with chronic non-cancer pain awaiting their first clinic appointment	728	No	SI	Logistic regression	51	61	35.0%	10, 15, 19
Samples, 2019[69]	USA	Cross-sectional	Nationwide questionnaire	Pts with and without opioid misuse	86,186	No	SB	Logistic regression	18-64	NA	N/A	13
Seo, 2019[70]	South Korea	Case-control	Multisite questionnaire	Pts with tension-type headache	332	No	SB	Logistic regression	47	67 (Case), 68 (Control)	24.7%	3, 7, 8, 12, 24, 25
Smith, 2004[71]	USA	Cross-sectional	Single site chart review, Multisite questionnaire	Pts with nonmalignant pain	153	No	SB	Logistic regression	45	57	19% (Passive SI), 13% (SI), 5% (Suicide plan), 5% (Previous SA)	16
Sun, 2020[c72]	China	Cross-sectional	Single site chart review, Single site questionnaire	Psychiatric outpatients with MDD	137	No	Past SI & SA	Logistic regression	28	62	22.6%	19
Tang, 2016[73]	UK	Cross-sectional	Single site questionnaire, Structured interview	Pts with chronic nonmalignant pain for 6 months or longer	62	No	SB	Hierarchical regression	52	68	22.6% (History SA)	15, 19
Tektonidou, 2011[c74]	USA	Cross-sectional	Nationwide questionnaire	Pts 40 years or older with arthritis, DM, or cancer	2,344	Yes	SI	Random forest model	62 (Arthritis), 62 (DM), 68 (Cancer)	60 (Arthritis), 52 (DM), 42 (Cancer)	5.6% (Arthritis), 6.8% (DM), 5.1% (Cancer)	1, 3, 5, 8, 16, 19, 24, 25, 26
Triñanes, 2015[75]	Spain	Cross-sectional	Multisite questionnaire	Women with FM	117	No	SI	Logistic regression	49	100	32.5%	16

Table S2. (Continued)

Author, year	Country	Study Design	Type of Data Sources	Study Population[a]	Total # Pts	Cancer Pts	Suicide-related Outcome(s)	Statistical methods	Age (Mean/ Median*)	Female %	Outcome Proportion	Identified Risk Factor Categories[b]
Tsai, 2019[76]	Taiwan	Retrospective cohort	National claims	Pts diagnosed with a fracture and matched controls	82,804 (Case), 82,804 (Control)	No	SB	Cox proportional hazards model	41	44	130/812,863 PY	7, 18, 24
Uebelacker, 2013[77]	USA	Prospective cohort	Multisite EMR, Multisite questionnaire, Structured interview	Pts with an anxiety disorder	676	No	SA	Cox proportional hazards model	41	67	6.0%	8, 11
Uğur, 2020[78]	Turkey	Cross-sectional	Multisite questionnaire	Pts with MDD	150	No	SA	Logistic regression	36 (Case), 34 (Control)	73 (Case), 68 (Control)	52.0%	8
Walker, 2008[79]	UK	Cross-sectional	Multisite questionnaire, Cancer registry	Cancer pts from an outpatient department	2,924	Yes	SI	Logistic regression	60	64	7.8%	1, 5, 16
Webb, 2012[80]	UK	Nested case-control	Multisite EMR	Primary care pts at risk of a first recorded episode of self-harm	2,306 (Case), 46,120 (Control)	Both	SB	Logistic regression	38*	45	N/A	1, 5, 17, 18, 23
Wilson, 2013[81]	Canada	Cross-sectional	Single site questionnaire	Rehabilitation pts with chronic musculoskeletal pain problems	303	No	SI	Multiple regression analysis	47	62	40.9%	16, 20
Wilson, 2017[82]	Canada	Cross-sectional	Single site questionnaire	Pts admitted to an outpatient chronic pain management program	282	No	SI	Logistic regression	48	70	31.9%	10, 11, 16, 20
Wolfe, 2011[83]	USA	Cross-sectional	Multisite questionnaire, Death certificates	Pts with FM recruited from community practice or registry databank	8,186	No	SD	Cox proportional hazards model	51	94	4.4%	9
Wolfe, 2020[84]	USA	Retrospective cohort	Multisite questionnaire, Death certificates	Pts with rheumatic diseases	35,248	No	SD	Cox proportional hazards model	62	81	0.4%	17
Xu, 2020[85]	China	Cross-sectional	Multisite questionnaire	Hospitalized cancer pts	544	Yes	SI	Logistic regression	Age 50–64: 52%	52	26.3%	16, 24
Zhang, 2020[86]	China	Cross-sectional	Multisite questionnaire	Pts diagnosed with cancer within 6 months	603	Yes	SI	Logistic regression	48	90	15.1%	3, 5, 8, 16, 18

Table S2. (Continued)

Author, year	Country	Study Design	Type of Data Sources	Study Population[a]	Total # Pts	Cancer Pts	Suicide-related Outcome(s)	Statistical methods	Age (Mean/ Median*)	Female %	Outcome Proportion	Identified Risk Factor Categories[b]
Zhong, 2017[87]	China	Cross-sectional	Multisite questionnaire, Multisite chart review	Cancer inpatients	517	Yes	SI	Logistic regression	60	51	15.3%	3, 6, 8, 17, 19

Abbreviations: **CH**: community healthy, **CP**: community patients, **CSA**: chronic short-acting, **DM**: diabetes mellitus, **EMR**: electronic medical record, **FM**: fibromyalgia, **GEE**: generalized estimating equations, **HC**: healthy controls, **LA**: long-acting, **LBP**: low-back pain, **LTOT**: long-term opioid therapy, **MANOVA**: multivariate analysis of variance, **MDD**: major depressive disorder, **MNSSA**: medically non-serious suicide attempt, **MSP**: multiple somatic pain, **MSSA**: medically serious suicide attempts, **NDI**: National Death Index, **N/A**: not available, **PC**: psychiatric controls, **pts**: patients, **PTSD**: post-traumatic stress disorder, **PY**: person-year, **RAP**: rehabilitation acute pain, **RCP**: rehabilitation chronic pain, **RM**: regular migraines, **RP**: rehabilitation patients without pain, **SA**: suicidal attempts, **SB**: suicidal behaviors, **SD**: suicide deaths, **SI**: suicidal ideations, **SM**: status migrainosus, **SSP**: single somatic pain, **SSV**: self-directed violence, **UK:** United Kingdom, **USA:** United States of America, **VHA**: Veterans Health Administration.

[a] All patients are adult patients (≥18 years old).

[b] We categorized individual risk factors into 27 risk factor categories including: (1) Age, (2) Antidepressant use and class, (3) Anxiety disorders and their severity, (4) Any pain medication use (non-opioids or unspecified), (5) Any unspecified physical health illness or comorbidity index, (6) Cancer, stages and related treatment (e.g., chemotherapy, surgery), (7) Cerebrovascular diseases or injuries, (8) Depression/depressive disorders and their severity, (9) Fibromyalgia, (10) Gender, (11) History of suicidal behavior/ ideation/attempts/suicidality, (12) Migraine /headaches and frequency, (13) Opioid use, dosage (e.g., >100 MME), type, ingredient, (14) Other medication use (e.g., phenobarbital), (15) Other mental health conditions, (16) Other patient reported factors (e.g., sexual/physical abuse, hopeless), (17) Other physical health conditions, (18) Other specific pain conditions, (19) Pain duration/severity/intensity, (20) Perceived burdensomeness, (21) PTSD, (22) Race/ethnicity, (23) Respiratory diseases, (24) Sleep disorders including insomnia, (25) Social determinants of health (SoDH), (26) Substance use disorder (SUD) or alcohol use disorder (AUD), and (27) Unspecific psychache/mental pain.

[c] Study that developed a suicide prediction model.

[d] SI related item: prefer death over disability.

Table S3. Summary of Individual Risk Factors Identified from Less Than 3 Studies by Data Source for Identification

**Risk factors**	**Data source that can be used to identify risk factors**
Each risk factor identified from 2 studies (2% of the 87 included studies)	
Dizziness, Family related issues, Household incomes/socioeconomic status, Pain avoidance, Perceived/feeling loneliness, Recent life events, Socialization issues	Unstructured/Collected data[a]
Any pain medication use, Diabetes, Fractures pain, Schizophrenia (e.g., schizoid tendencies)	Structured
Aggression/impulsivity, Domestic violence experience or witness, Education level, Personality or borderline personality disorders, Unspecified SUD (not including AUD)	Structured/Unstructured/Collected data
Each risk factor identified from only 1 study (1% of the 87 included studies)	
Bad habits, Binges duration, Caregiver issues, Childhood adversity experience, Chronic maladjustment, Committed action, Daily exercise time, Disability, Doctor Dissatisfaction, Fatigue, General acceptance, Lack of appetite, Lost-time injuries, Low health self-efficacy, Number of close friends, Other personal feelings, Perceived/feeling helpless, Pericranial tenderness, Physical functioning, Poor functional status, Religion/spirituality, Revenge, Self-blame, Self-confidence, Self-disclosure, Smoking, Socially withdrawn, Somatic complaints, SM, Substance abuse, Symptom dependency, Weakness	Unstructured/Collected data
Asthma, Bipolar disorder, Cardiovascular diseases, Emotional detachment, Epilepsy, Insurance priority, Liver diseases, Male genital disorders, Mood disorders, Opioid ingredients (e.g., oxycodone) and type, Chest pain, Musculoskeletal pain, Neck/shoulder pain, TMJ pain, Phenobarbital use, Physical illness, Psychiatric disorders, Rheumatic diseases, Stroke, TBI	Structured
Cannabis use problems, Cognitive confusion, Family history of depression, Family history of suicide/suicide attempts, Postoperative complications, Tinnitus	Structured/Unstructured/Collected data

Abbreviations: **AUD:** alcohol use disorder, **SUD:** Substance use disorder, **SM:** Status migrainosus**, TBI:** traumatic brain injury, **TMJ:** temporomandibular disorders.

[a] We categorized the type of data sources that can be used to identify and measure each risk factor into: 1) “structured data” that naturally occur (e.g., as a result of clinical documentation or billing activities) and outside a research context such as structured EMR or administrative claims data; 2) “unstructured data” include unstructured clinical notes in EMR required efforts such as natural language processing to extract information; and 3) “collected data” that require additional design such as from a questionnaire or registry. Structured/Unstructured/Collected data refers to some of the risk factors (e.g., depression diagnosis) may be identified from structured data, and some (e.g., depression severity) may be identified from unstructured data or questionnaire.

Table S4. Identified Risk Factors in Each Risk Factor Category

Risk Factor Category	Individual Risk Factors
Age	Different age thresholds
Antidepressant use and type	Antidepressant use and type
Anxiety disorders and their severity	Anxiety disorders and severity
Any pain medication use (non-opioids or unspecified)	Any pain medication use
Any unspecified physical health illness or comorbidity index	Unspecified physical health illness, Comorbidity or comorbidity index, Any unspecified physical or somatic pain conditions, Physical illness
Cancer, stages and related treatment (e.g., chemotherapy, surgery)	Cancer, stages and related treatment (e.g., chemotherapy, surgery)
Cerebrovascular diseases or injuries	Pericranial tenderness, Stroke, TBI
Depression/depressive disorders and their severity	Depression/depressive disorders and severity
Fibromyalgia	Fibromyalgia pain
Gender	Gender
History of suicidal behavior/ideation/attempts/suicidality	History of suicidal behavior/ideation/attempts/suicidality
Migraine /headaches and frequency	Pain condition: migraine /headaches and frequency, SM
Opioid use, dosage (e.g., >100 MME), type, ingredient	Opioid ingredients (e.g., oxycodone) and type, Opioid use and dosage (e.g., >100 MME)
Other medication use (e.g., phenobarbital)	Phenobarbital use
Other mental health conditions	Aggression/impulsivity, Anger issues, Any mental health illness, Bipolar disorder, Emotional detachment, Mental quality of life, Mood disorders, Personality or boaderline personality disorders, Psychiatric disorders, Schizophrenia (e.g., schizoid tendencies)
Other patient reported factors (e.g., sexual/physical abuse, hopeless)	Bad habits, Binges duration, Caregiver issues, Childhood adversity experience, Chronic maladjustment, Committed action, Daily exercise time, Disability, Dizziness, Doctor Dissatisfaction, Domestic violence experience or witness, Family history of depression, Family history of suicide/suicide attempts, Family related issues, Fatigue, General acceptance, History of sexual/physical abuse, Insurance priority, Lack of appetite, Lost-time injuries, Low health self-efficacy, Mental quality of life, Number of close friends, Other personal feelings, Perceived/feeling helpless, Perceived/feeling hopeless, Perceived/feeling loneliness, Perceived/feeling stressful, Personality or borderline personality disorders, Physical functioning, Recent life events, Revenge, Religion/spirituality, Self-blame, Self-confidence, Self-disclosure, Smoking, Socialization issues, Socially withdrawn, Somatic complaints, Substance abuse, Symptom dependency, Weakness
Other physical health conditions	Diabetes, Epilepsy, Liver diseases, Cardiovascular diseases, Cognitive confusion, Male genital disorders, Poor functional status, Postoperative complications, Rheumatic diseases, Tinnitus
Other specific pain conditions	Cancer pain, Chest pain, Back pain/low back pain, Fractures pain, Musculoskeletal pain, Neck/shoulder pain, TMJ pain
Pain duration/severity/intensity	Pain avoidance, Pain catastrophizing, Pain duration/severity/intensity
Perceived burdensomeness	Perceived burdensomeness
PTSD	PTSD
Race/ethnicity	Race/ethnicity
Respiratory diseases	Asthma, Respiratory diseases

Sleep disorders including insomnia	Sleep disorders including insomnia
Social determinants of health	Household incomes/socioeconomic status, Marital status (e.g., unmarried), Unemployment
SUD/AUD	AUD, Cannabis use problems, Drug use disorders, SUD (not including AUD)
Unspecific psychache/mental pain	Psychache/mental pain

Abbreviations: **AUD:** alcohol use disorder, **MME:** morphine milligram equivalents, **PTSD:** Posttraumatic stress disorder, **SUD:** Substance use disorder, **SM:** Status migrainosus**, TBI:** traumatic brain injury, **TMJ:** temporomandibular disorders.

References:

1. Abdelghani M, Ibrahim S, Said A, Foad E. Can Prescription Drug Use Disorder Predict Suicidality in US Adults With Chronic Pain? A Pilot Study Based on Collaborative Psychiatric Epidemiological Surveys. *Journal of addiction medicine*. 2020;14(6):e330-e336. doi:10.1097/ADM.0000000000000670
2. Akechi T, Okamura H, Nishiwaki Y, Uchitomi Y. Predictive factors for suicidal ideation in patients with unresectable lung carcinoma: A 6-month follow-up study. *Cancer*. 2002;95(5):1085-1093. doi:10.1002/cncr.10769
3. Akechi T, Okuyama T, Uchida M, et al. Factors associated with suicidal ideation in patients with multiple myeloma. *Japanese journal of clinical oncology*. 2020;doi:10.1093/jjco/hyaa143
4. Alacreu-Crespo A, Cazals A, Courtet P, Olié E. Brief assessment of psychological pain to predict suicidal events at one year in depressed patients. *Psychotherapy and Psychosomatics*. 2020;89(5):320-323. doi:10.1159/000506957
5. Andrijic NL, Alajbegovic A, Zec SL, Loga S. SUICIDAL IDEATION AND THOUGHTS OF DEATH IN EPILEPSY PATIENTS. *Psychiatr Danub*. 2014;26(1):52-55.
6. Applebaum KM, Asfaw A, O'Leary PK, Busey A, Tripodis Y, Boden LI. Suicide and drug-related mortality following occupational injury. *American Journal of Industrial Medicine*. 2019;62(9):733-741. doi:10.1002/ajim.23021
7. Ashrafioun L, Kane C, Stephens B, Britton PC, Conner KR. Suicide attempts among alcohol-dependent pain patients before and after an inpatient hospitalization. *Drug and Alcohol Dependence*. 2016;163:209-215. doi:10.1016/j.drugalcdep.2016.04.024
8. Ashrafioun L, Kane C, Bishop TM, Britton PC, Pigeon WR. The Association of Pain Intensity and Suicide Attempts Among Patients Initiating Pain Specialty Services. *Journal of Pain*. 2019;20(7):852-859. doi:10.1016/j.jpain.2019.01.012
9. Berhane HY, Jamerson-Dowlen B, Friedman LE, Berhane Y, Williams MA, Gelaye B. Association between migraine and suicidal behavior among Ethiopian adults. *BMC Psychiatry*. 2018;18:9. doi:10.1186/s12888-018-1629-7
10. Blakey SM, Wagner HR, Naylor J, et al. Chronic Pain, TBI, and PTSD in Military Veterans: A Link to Suicidal Ideation and Violent Impulses? *Journal of Pain*. 2018;19(7):797-806. doi:10.1016/j.jpain.2018.02.012
11. Braden JB, Sullivan MD. Suicidal Thoughts and Behavior Among Adults With Self-Reported Pain Conditions in the National Comorbidity Survey Replication. *Journal of Pain*. 2008;9(12):1106-1115. doi:10.1016/j.jpain.2008.06.004
12. Breslau N, Schultz L, Lipton R, Peterson E, Welch KM. Migraine headaches and suicide attempt. *Headache*. 2012;52(5):723-31. doi:10.1111/j.1526-4610.2012.02117.x
13. Brown LA, Lynch KG, Cheatle M. Pain catastrophizing as a predictor of suicidal ideation in chronic pain patients with an opiate prescription. *Psychiatry Research*. 2020;286doi:10.1016/j.psychres.2020.112893
14. Bryant RA, O'Donnell ML, Forbes D, McFarlane AC, Silove D, Creamer M. The course of suicide risk following traumatic injury. *Journal of Clinical Psychiatry*. 2016;77(5):648-653. doi:10.4088/JCP.14m09661
15. Caceda R, Durand D, Cortes E, et al. Impulsive Choice and Psychological Pain in Acutely Suicidal Depressed Patients. *Psychosom Med*. 2014;76(6):445-451. doi:10.1097/psy.0000000000000075
16. Calandre EP, Navajas-Rojas MA, Ballesteros J, Garcia-Carrillo J, Garcia-Leiva JM, Rico-Villademoros F. Suicidal ideation in patients with fibromyalgia: a cross-sectional study. *Pain practice : the official journal of World Institute of Pain*. 2015;15(2):168-74. doi:10.1111/papr.12164
17. Campbell G, Bruno R, Darke S, Degenhardt L. Associations of borderline personality with pain, problems with medications and suicidality in a community sample of chronic non-cancer pain patients prescribed opioids for pain. *General Hospital Psychiatry*. 2015;37(5):434-440. doi:10.1016/j.genhosppsych.2015.05.004
18. Campbell G, Bruno R, Darke S, et al. Prevalence and correlates of suicidal thoughts and suicide attempts in people prescribed pharmaceutical opioids for chronic pain. *Clinical Journal of Pain*. 2016;32(4):292-301. doi:10.1097/AJP.0000000000000283
19. Campbell G, Darke S, Degenhardt L, et al. Prevalence and Characteristics Associated with Chronic Noncancer Pain in Suicide Decedents: A National Study. *Suicide Life-Threat Behav*.14. doi:10.1111/sltb.12627

20. Cheatle MD, Wasser T, Foster C, Olugbodi A, Bryan J. Prevalence of suicidal ideation in patients with chronic non-cancer pain referred to a behaviorally based pain program. *Pain Physician*. 2014;17(3):E359-E367.
21. Copeland LA, McIntyre RT, Stock EM, Zeber JE, MacCarthy DJ, Pugh MJ. Prevalence of suicidality among hispanic and African American veterans following surgery. *American Journal of Public Health*. 2014;104(SUPPL. 4):S603-S608. doi:10.2105/AJPH.2014.301938
22. Demidenko MI, Dobscha SK, Morasco BJ, Meath THA, Ilgen MA, Lovejoy TI. Suicidal ideation and suicidal self-directed violence following clinician-initiated prescription opioid discontinuation among long-term opioid users. *General Hospital Psychiatry*. 2017;47:29-35. doi:10.1016/j.genhosppsych.2017.04.011
23. Edwards RR, Smith MT, Kudel I, Haythornthwaite J. Pain-related catastrophizing as a risk factor for suicidal ideation in chronic pain. *Pain*. 2006;126(1-3):272-279. doi:10.1016/j.pain.2006.07.004
24. Edwards RR, Magyar-Russell G, Thomb G, et al. Acute pain at discharge from hospitalization is a prospective predictor of long-term suicidal ideation after burn injury. *Archives of Physical Medicine & Rehabilitation*. 2007;88:S36-42. doi:10.1016/j.apmr.2007.05.031
25. Fishbain DA, Bruns D, Disorbio JM, Lewis JE. Risk for five forms of suicidality in acute pain patients and chronic pain patients vs pain-free community controls. *Pain Medicine*. 2009;10(6):1095-1105. doi:10.1111/j.1526-4637.2009.00682.x
26. Fishbain DA, Bruns D, Lewis JE, Disorbio JM, Gao J, Meyer LJ. Predictors of Homicide-Suicide Affirmation in Acute and Chronic Pain Patients. *Pain Medicine*. 2011;12(1):127-137. doi:10.1111/j.1526-4637.2010.01013.x
27. Fishbain DA, Bruns D, Meyer LJ, Lewis JE, Gao J, Disorbio JM. Exploration of the relationship between disability perception, preference for death over disability, and suicidality in patients with acute and chronic pain. *Pain medicine (Malden, Mass)*. 2012;13(4):552-61. doi:10.1111/j.1526-4637.2012.01358.x
28. Fishbain DA, Lewis JE, Bruns D, Gao J, Disorbio JM, Meyer L. Patient predictor variables for six forms of suicidality. *European journal of pain (London, England)*. 2012;16(5):706-717. doi:10.1002/j.1532-2149.2011.00033.x
29. Fishbain DA, Bruns A, Gao JR, Lewis JE, Meyer LJ, Disorbio JM. The Perception of Being a Burden in Acute and Chronic Pain Patients Is Associated with Affirmation of Different Types of Suicidality. *Pain Med*. 2016;17(3):530-538. doi:10.1111/pme.12889
30. Fonda JR. *Association of traumatic brain injury with intentional and unintentional injury among United States Operation Enduring Freedom, Operation Iraqi Freedom and Operation New Dawn veterans*. Boston University; 2015. https://login.lp.hscl.ufl.edu/login?url=https://www.proquest.com/dissertations-theses/association-traumatic-brain-injury-with/docview/1728037588/se-2?accountid=10920http://resolver.ebscohost.com/openurl?ctx_ver=Z39.88-2004&ctx_enc=info:ofi/enc:UTF-8&rfr_i
31. Frumkin MR, Robinaugh DJ, LeBlanc NJ, et al. The pain of grief: Exploring the concept of psychological pain and its relation to complicated grief, depression, and risk for suicide in bereaved adults. *J Clin Psychol*.14. doi:10.1002/jclp.23024
32. Fuller-Thomson E, Hodgins GA. Suicide Attempts among Those with Migraine: Findings from a Nationally Representative Canadian Study. *Arch Suicide Res*. 2020;24:360-379. doi:10.1080/13811118.2019.1578710
33. Gensichen J, Teising A, Konig J, Gerlach FM, Petersen JJ. Predictors of suicidal ideation in depressive primary care patients. *J Affect Disord*. 2010;125(1-3):124-127. doi:10.1016/j.jad.2009.12.008
34. Harnod T, Lin CL, Kao CH. Risk and Predisposing Factors for Suicide Attempts in Patients with Migraine and Status Migrainosus: A Nationwide Population-Based Study. *J Clin Med*. 2018;7(9):11. doi:10.3390/jcm7090269
35. Hashash JG, Vachon A, Ramos Rivers C, et al. Predictors of Suicidal Ideation Among IBD Outpatients. *Journal of clinical gastroenterology*. 2019;53(1):e41-e45. doi:10.1097/mcg.0000000000001094
36. Ilgen MA, Zivin K, Austin KL, et al. Severe pain predicts greater likelihood of subsequent suicide. *Suicide & life-threatening behavior*. 2010;40(6):597-608. doi:10.1521/suli.2010.40.6.597
37. Ilgen MA, Kleinberg F, Ignacio RV, et al. Noncancer pain conditions and risk of suicide. *JAMA psychiatry*. 2013;70(7):692-7. doi:10.1001/jamapsychiatry.2013.908

38. Ilgen MA, Bohnert ASB, Ganoczy D, Bair MJ, McCarthy JF, Blow FC. Opioid dose and risk of suicide. *Pain*. 2016;157(5):1079-1084. doi:10.1097/j.pain.0000000000000484
39. Im JJ, Shachter RD, Oliva EM, Henderson PT, Paik MC, Trafton JA. Association of Care Practices with Suicide Attempts in US Veterans Prescribed Opioid Medications for Chronic Pain Management. *Journal of General Internal Medicine*. 2015;30(7):979-991. doi:10.1007/s11606-015-3220-y
40. Jakubczyk A, Ashrafioun L, Ilgen M, et al. Physical Pain and History of Suicidal Behaviors in Alcohol-Dependent Patients Entering Treatment in Poland. *Subst Use Misuse*. 2016;51(10):1307-1317. doi:10.3109/10826084.2016.1168444
41. Jang SY, Cha Y, Kwak JH, Kim KJ, Kim HY, Choy WS. What Is the Difference in the Risk of Suicide Death Between Spine Fracture in Patients Older Than 65 Years and Matched Controls? A Large-database Study from South Korea. *Clinical orthopaedics and related research*. 2020;478(11):2422-2430. doi:10.1097/CORR.0000000000001414
42. Jeon HJ, Woo JM, Kim HJ, et al. Gender differences in somatic symptoms and current suicidal risk in outpatients with major depressive disorder. *Psychiatry Investigation*. 2016;13(6):609-615. doi:10.4306/pi.2016.13.6.609
43. Jha MK, Fava M, Minhajuddin A, et al. Association of anger attacks with suicidal ideation in adults with major depressive disorder: Findings from the EMBARC study. *Depression and Anxiety*. 2020;doi:10.1002/da.23095
44. Jimenez-Rodriguez I, Garcia-Leiva JM, Jimenez-Rodriguez BM, Condes-Moreno E, Rico-Villademoros F, Calandre EP. Suicidal ideation and the risk of suicide in patients with fibromyalgia: a comparison with non-pain controls and patients suffering from low-back pain. *Neuropsychiatr Dis Treat*. 2014;10:625-630. doi:10.2147/ndt.557596
45. Johnson CC, Phillips KM, Miller SN. Suicidal Ideation among Veterans Living with Cancer Referred to Mental Health. *Clin Gerontol*. 2020;43(1):24-36. doi:10.1080/07317115.2019.1686719
46. Kanzler KE, Bryan CJ, McGeary DD, Morrow CE. Suicidal Ideation and Perceived Burdensomeness in Patients with Chronic Pain. *Pain Practice*. 2012;12(8):602-609. doi:10.1111/j.1533-2500.2012.00542.x
47. Lan CC, Tseng CH, Chen JH, et al. Increased risk of a suicide event in patients with primary fibromyalgia and in fibromyalgia patients with concomitant comorbidities A nationwide population-based cohort study. *Medicine (Baltimore)*. 2016;95(44):9. doi:10.1097/md.0000000000005187
48. Levi-Belz Y, Gvion Y, Horesh N, et al. Mental Pain, Communication Difficulties, and Medically Serious Suicide Attempts: A Case-Control Study. *Archives of Suicide Research*. 2014;18(1):74-87. doi:10.1080/13811118.2013.809041
49. Levi-Belz Y, Gvion Y, Grisaru S, Apter A. When the Pain Becomes Unbearable: Case-Control Study of Mental Pain Characteristics Among Medically Serious Suicide Attempters. *Archives of Suicide Research*. 2018;22(3):380-393. doi:10.1080/13811118.2017.1355288
50. Levi-Belz Y, Gvion Y, Levi U, Apter A. Beyond the mental pain: A case-control study on the contribution of schizoid personality disorder symptoms to medically serious suicide attempts. *Comprehensive Psychiatry*. 2019;90:102-109. doi:10.1016/j.comppsych.2019.02.005
51. Li H, Xie W, Luo X, et al. Clarifying the role of psychological pain in the risks of suicidal ideation and suicidal acts among patients with major depressive episodes. *Suicide and Life-Threatening Behavior*. 2014;44(1):78-88. doi:10.1111/sltb.12056
52. Liu HY, Fuh JL, Lin YY, Chen WT, Wang SJ. Suicide risk in patients with migraine and comorbid fibromyalgia. *Neurology*. 2015;85(12):1017-1023. doi:10.1212/wnl.0000000000001943
53. Lopez-Morinigo J-D, Fernandes AC, Shetty H, et al. Can risk assessment predict suicide in secondary mental healthcare? Findings from the South London and Maudsley NHS Foundation Trust Biomedical Research Centre (SLaM BRC) Case Register. *Social Psychiatry and Psychiatric Epidemiology*. 2018/11/01 2018;53(11):1161-1171. doi:10.1007/s00127-018-1536-8
54. Margari F, Lorusso M, Matera E, et al. Aggression, Impulsivity, And suicide risk in benign chronic pain patients – a cross-sectional study. *Neuropsychiatric Disease and Treatment*. 2014;10:1613-1620. doi:10.2147/NDT.S66209

55. Mazereeuw G, Gomes T, Macdonald EM, et al. Oxycodone, Hydromorphone, and the Risk of Suicide: A Retrospective Population-Based Case-Control Study. *Drug Saf*. 2020;43(8):737-743. doi:10.1007/s40264-020-00924-5
56. McCracken LM, Patel S, Scott W. The role of psychological flexibility in relation to suicidal thinking in chronic pain. *European Journal of Pain (United Kingdom)*. 2018;22(10):1774-1781. doi:10.1002/ejp.1273
57. McKernan LC, Lenert MC, Crofford LJ, Walsh CG. Outpatient Engagement and Predicted Risk of Suicide Attempts in Fibromyalgia. *Arthritis Care & Research*. 2019;71(9):1255-1263. doi:https://doi.org/10.1002/acr.23748
58. Mitchell R, Draper B, Harvey L, Brodaty H, Close J. The association of physical illness and self-harm resulting in hospitalisation among older people in a population-based study. *Aging Ment Health*. 2017;21(3):279-288. doi:10.1080/13607863.2015.1099610
59. Noyman-Veksler G, Lerman SF, Joiner TE, et al. Role of Pain-Based Catastrophizing in Pain, Disability, Distress, and Suicidal Ideation. *Psychiatry (New York)*. 2017;80(2):155-170. doi:10.1080/00332747.2016.1230984
60. Orr MF, Rogers AH, Shepherd JM, et al. Is there a relationship between cannabis use problems, emotion dysregulation, and mental health problems among adults with chronic pain? *Psychol Health Med*. 2020;25(6):742-755. doi:10.1080/13548506.2019.1653485
61. Ozdemiroglu F, Memis CO, Meydan N, et al. Self-Esteem, Pain and Suicidal Thoughts in a Sample of Cancer Patients. *J Mood Disord*. 2017;7(3):156-162. doi:10.5455/jmood.20170804091851
62. Panagioti M, Gooding PA, Tarrier N. Hopelessness, defeat, and entrapment in posttraumatic stress disorder: Their association with suicidal behavior and severity of depression. *Journal of Nervous and Mental Disease*. 2012;200(8):676-683. doi:10.1097/NMD.0b013e3182613f91
63. Park S, Cho MJ, Seong S, et al. Psychiatric morbidities, sleep disturbances, suicidality, and quality-of-life in a community population with medically unexplained pain in Korea. *Psychiatry Research*. 2012;198(3):509-515. doi:10.1016/j.psychres.2012.01.028
64. Park SA, Chung SH, Lee Y. Factors associated with suicide risk in advanced cancer patients: A cross-sectional study. *Asian Pacific Journal of Cancer Prevention*. 2016;17(11):4831-4836. doi:10.22034/APJCP.2016.17.11.4831
65. Park MJ, Choi KW, Na EJ, et al. Multiple types of somatic pain increase suicide attempts and depression: A nationwide community sample of Korean adults. *Comprehensive Psychiatry*. 2019;90:43-48. doi:10.1016/j.comppsych.2018.12.006
66. Park S, Heo HA, Yun KI, Pyo SW. High prevalence of stress and suicidal ideation in women with temporomandibular disorder: A population-based cross-sectional survey. *Cranio-J Craniomandib Sleep Pract*.7. doi:10.1080/08869634.2020.1721174
67. Racine M, Choinière M, Nielson WR. Predictors of suicidal ideation in chronic pain patients: An exploratory study. *Clinical Journal of Pain*. 2014;30(5):371-378. doi:10.1097/AJP.0b013e31829e9d4d
68. Racine M, Sánchez-Rodríguez E, Gálan S, et al. Factors associated with suicidal ideation in patients with chronic non-cancer pain. *Pain Medicine (United States)*. 2017;18(2):283-293. doi:10.1093/pm/pnw115
69. Samples H, Stuart EA, Olfson M. Opioid Use and Misuse and Suicidal Behaviors in a Nationally Representative Sample of US Adults. *Am J Epidemiol*. 2019;188(7):1245-1253. doi:10.1093/aje/kwz061
70. Seo JG, Kim KT, Moon HJ, Kuk Do J, Kim SY, Park SP. Suicidality and its risk factors in tension-type headache patients: A multicenter case-control study. *Journal of Clinical Neuroscience*. 2019;69:21-25. doi:10.1016/j.jocn.2019.08.084
71. Smith MT, Edwards RR, Robinson RC, Dworkin RH. Suicidal ideation, plans, and attempts in chronic pain patients: Factors associated with increased risk. *Pain*. 2004;111(1-2):201-208. doi:10.1016/j.pain.2004.06.016
72. Sun X, Li H, Song W, Jiang S, Shen C, Wang X. ROC analysis of three-dimensional psychological pain in suicide ideation and suicide attempt among patients with major depressive disorder. *Journal of clinical psychology*. 2020;76(1):210-227. doi:10.1002/jclp.22870
73. Tang NK, Beckwith P, Ashworth P. Mental Defeat Is Associated With Suicide Intent in Patients With Chronic Pain. *The Clinical journal of pain*. 2016;32(5):411-9. doi:10.1097/ajp.0000000000000276

74. Tektonidou MG, Dasgupta A, Ward MM. Suicidal ideation among adults with arthritis: prevalence and subgroups at highest risk. Data from the 2007-2008 National Health and Nutrition Examination Survey. *Arthritis care & research*. 2011;63(9):1322-33. doi:10.1002/acr.20516
75. Triñanes Y, González-Villar A, Gómez-Perretta C, Carrillo-de-la-Peña MT. Suicidality in Chronic Pain: Predictors of Suicidal Ideation in Fibromyalgia. *Pain Practice*. 2015;15(4):323-332. doi:10.1111/papr.12186
76. Tsai CH, Cheng WJ, Muo CH, Lin TL. Fractures as a suicidal behavior risk factor A nationwide population-based cohort study. *Medicine (Baltimore)*. 2019;98(3):5. doi:10.1097/md.0000000000014148
77. Uebelacker LA, Weisberg R, Millman M, Yen S, Keller M. Prospective study of risk factors for suicidal behavior in individuals with anxiety disorders. *Psychological Medicine*. 2013;43(7):1465-1474. doi:10.1017/S0033291712002504
78. Uğur K, Demirkol ME, Tamam L. The Mediating Roles of Psychological Pain and Dream Anxiety in the Relationship between Sleep Disturbance and Suicide. *Archives of suicide research : official journal of the International Academy for Suicide Research*. 2020:1-17. doi:10.1080/13811118.2020.1740124
79. Walker J, Waters RA, Murray G, et al. Better off dead: Suicidal thoughts in cancer patients. *Journal of Clinical Oncology*. 2008;26(29):4725-4730. doi:10.1200/JCO.2007.11.8844
80. Webb RT, Kontopantelis E, Doran T, Qin P, Creed F, Kapur N. Risk of self-harm in physically ill patients in UK primary care. *J Psychosomat Res*. 2012;73(2):92-97. doi:10.1016/j.jpsychores.2012.05.010
81. Wilson KG, Kowal J, Henderson PR, McWilliams LA, Péloquin K. Chronic pain and the interpersonal theory of suicide. *Rehabilitation Psychology*. 2013;58(1):111-115. doi:10.1037/a0031390
82. Wilson KG, Heenan A, Kowal J, Henderson PR, McWilliams LA, Castillo D. Testing the Interpersonal Theory of Suicide in Chronic Pain. *Clinical Journal of Pain*. 2017;33(8):699-706. doi:10.1097/AJP.0000000000000451
83. Wolfe F, Hassett AL, Walitt B, Michaud K. Mortality in fibromyalgia: A study of 8,186 patients over thirty-five years. *Arthritis Care & Research*. 2011;63(1):94-101. doi:https://doi.org/10.1002/acr.20301
84. Wolfe F, Ablin J, Baker JF, et al. All-cause and cause-specific mortality in persons with fibromyalgia and widespread pain: An observational study in 35,248 persons with rheumatoid arthritis, non-inflammatory rheumatic disorders and clinical fibromyalgia. *Seminars in Arthritis and Rheumatism*. 2020;doi:10.1016/j.semarthrit.2020.02.005
85. Xu Q, Jia S, Fukasawa M, et al. A cross-sectional study on associations of physical symptoms, health self-efficacy, and suicidal ideation among Chinese hospitalized cancer patients. *BMC Psychiatry*. 2020;20(1)doi:10.1186/s12888-020-02945-x
86. Zhang Y, Li W, Zhang Z, et al. Suicidal Ideation in Newly-Diagnosed Chinese Cancer Patients. *Frontiers in Psychiatry*. 2020;11doi:10.3389/fpsyt.2020.00708
87. Zhong BL, Li SH, Lv SY, et al. Suicidal ideation among Chinese cancer inpatients of general hospitals: Prevalence and correlates. *Oncotarget*. 2017;8(15):25141-25150. doi:10.18632/oncotarget.15350
